# Rat Pharmacokinetics and In Vitro Metabolite Identification of KM-819, a Parkinson’s Disease Candidate, Using LC-MS/MS and LC-HRMS

**DOI:** 10.3390/molecules29051004

**Published:** 2024-02-25

**Authors:** Hae-In Choi, Taeheon Kim, Jin Woo Kim, Gi Ju Lee, Jinyoung Choi, Yoon-Jee Chae, Eunhee Kim, Tae-Sung Koo

**Affiliations:** 1 Graduate School of New Drug Discovery and Development, Chungnam National University, Daejeon 34134, Republic of Korea; chi705@naver.com (H.-I.C.); thkim150@daewoong.co.kr (T.K.); dpslzk333@naver.com (J.W.K.); dlrlwn1234@naver.com (G.J.L.); jinyoung724@naver.com (J.C.); 2 College of Pharmacy, Woosuk University, Wanju 55338, Republic of Korea; yjchae@woosuk.ac.kr; 3 College of Biological Sciences and Biotechnology, Chungnam National University, Daejeon 34134, Republic of Korea; eunhee@cnu.ac.kr; 4 Biopharmaceutical Division, Kainos Medicine Inc., Seongnam 13488, Republic of Korea

**Keywords:** KM-819, FAF1 inhibitor, LC-MS/MS, LC-HRMS, pharmacokinetics, metabolite identification

## Abstract

FAF1 (FAS-associated factor 1) is involved in the activation of Fas cell surface death receptors and plays a role in apoptosis and necrosis. In patients with Parkinson’s disease, FAF1 is overexpressed in dopaminergic neurons in the substantia nigra. KM-819, an FAF1 inhibitor, has shown potential for preventing dopaminergic neuronal cell death, promoting the degradation of α-synuclein and preventing its accumulation. This study aimed to develop and validate a quantitative analytical method for determining KM-819 levels in rat plasma using liquid chromatography–tandem mass spectrometry. This method was then applied to pharmacokinetic (PK) studies in rats. The metabolic stability of KM-819 was assessed in rat, dog, and human hepatocytes. In vitro metabolite identification and metabolic pathways were investigated in rat, dog, and human hepatocytes. The structural analog of KM-819, namely *N*-[1-(4-bromobenzyl)-3,5-dimethyl-1*H*-pyrazol-4-yl]-2-(phenylsulfanyl) acetamide, served as the internal standard (IS). Proteins were precipitated from plasma samples using acetonitrile. Analysis was carried out using a reverse-phase C18 column with a mobile phase consisting of 0.1% formic acid in distilled water and 0.1% formic acid in acetonitrile. The analytical method developed for KM-819 exhibited linearity within the concentration range of 0.002–10 μg/mL in rat plasma. The precision and accuracy of the intra- and inter-day measurements were <15% for the lower limit of quantification (LLOQ) and all quality control samples. KM-819 demonstrated stability under various sample storage conditions (6 h at room temperature (25 °C), four weeks at −20 °C, three freeze-thaw cycles, and pretreated samples in the autosampler). The matrix effect and dilution integrity met the criteria set by the Food and Drug Administration and the European Medicines Agency. This sensitive, rapid, and reliable analytical method was successfully applied in pharmacokinetic studies in rats. Pharmacokinetic analysis revealed the dose-independent kinetics of KM-819 at 0.5–5 mg/kg, with a moderate oral bioavailability of ~20% in rats. The metabolic stability of KM-819 was also found to be moderate in rat, dog, and human hepatocytes. Metabolite identification in rat, dog, and human hepatocytes resulted in the discovery of six, six, and eight metabolites, respectively. Glucuronidation and mono-oxidation have been proposed as the major metabolic pathways. Overall, these findings contribute to a better understanding of the pharmacokinetic characteristics of KM-819, thereby aiding future clinical studies.

## 1. Introduction

Parkinson’s disease (PD) is a prominent neurodegenerative disorder characterized by tremors, muscle rigidity, bradykinesia (slow movement), and postural instability [1]. PD is pathologically marked by the selective death of dopaminergic neurons in the substantia nigra pars compacta (SNpc) of the midbrain and by the accumulation of abnormal protein aggregates, known as Lewy bodies, within the brain [1,2]. Lewy bodies, which are primarily composed of misfolded and aggregated α-synuclein (α-syn) proteins, are also observed in other neurodegenerative conditions such as Lewy body dementia (LBD) and multiple system atrophy (MSA). The α-syn protein is predominantly located at presynaptic terminals and plays a key role in the regulation of the synaptic endoplasmic reticulum and dopamine release [3]. Under normal conditions, α-syn exists as a monomer; however, due to gene mutations or abnormal abundance, the protein can misfold and aggregate into toxic oligomers or fibrils, impairing regular dopamine release into synapses and consequently contributing to PD pathogenesis [4].

Several medications, including levodopa, anticholinergics, monoamine oxidase-B (MAO-B) inhibitors, and dopamine receptor agonists, are currently used to manage PD. Although these drugs can alleviate symptoms, they fail to halt disease progression or provide a cure [5]. Moreover, their effectiveness decreases over time, necessitating increased doses, which can lead to severe side effects, such as dyskinesia, psychiatric disorders, and sleep problems [6]. With the increasing prevalence of age-related disorders owing to a longer lifespan, the number of PD cases is growing annually, underscoring the urgent need for more effective therapeutic strategies.

Fas-associated factor 1 (FAF1), a protein that promotes cell death pathways including apoptosis and necrosis, is a novel and unexplored target for drug development. FAF1 is uniquely overexpressed in SNpc dopaminergic neurons in patients with PD, fostering α-syn accumulation through autophagic suppression and encouraging dopaminergic neuronal cell death [7,8,9]. Thus, FAF1 is considered to be a significant contributor to the etiology and progression of PD.

KM-819 (or KR-33493), which is under development by Kainos Medicine, Inc. (Seongnam, Korea), is a pioneering FAF1 inhibitor and candidate for PD and MSA treatment and has been demonstrated to show potential for inhibiting dopaminergic neuronal cell death and activating α-syn degradation via autophagy. Previous studies have shown that KM-819 protects striatal dopamine neurons in 1-methyl-4-phenyl-1,2,3,6-tetrahydropyridine (MPTP) mice and decreases α-syn accumulation in mouse midbrains that overexpress FAF1 [10,11]. Moreover, KM-819 caused no adverse effects following repeated administration in Sprague-Dawley (SD) rats for four weeks (up to 500 mg/kg/day) or in beagle dogs for two weeks (up to 1000 mg/kg/day) [12]. In addition, a Phase 1 clinical trial in 2018 on healthy Korean subjects reported no serious adverse effects [13] and phase 2 clinical studies on Parkinson’s disease are currently underway in the United States [14]. Furthermore, KM-819 has been approved for phase 2 clinical trials on MSA in Korea [15].

Although KM-819 has been the subject of numerous studies, there is a scarcity of information regarding its pharmacokinetic properties in rodents and suitable analytical methods for its measurement in biological samples. As one example of a previous study, Jeong et al. [12] reported the plasma exposure pattern of KM-819 during a repeated rat toxicity test but did not provide detailed plasma concentrations, pharmacokinetic parameters, or specifics regarding the analytic method. Similarly, Shin et al. [13] used liquid chromatography–tandem mass spectrometry (LC-MS/MS) to analyze the plasma concentrations of KM-819 after oral administration in a Phase 1 clinical trial; however, the detailed analytical methodology remains unknown.

Thus, in the present study, we propose a simple and rapid LC-MS/MS method for detecting KM-819 in rat plasma. This method is validated in compliance with the Food and Drug Administration (FDA) and European Medicines Agency (EMA) guidelines [16,17] and is applied to pharmacokinetic studies in rats. The rat pharmacokinetic studies are conducted at three different doses to investigate the dose-dependency of KM-819. In addition, the metabolic stability of KM-819 in rat, dog, and human hepatocytes is assessed and human clearance is predicted using an in vitro-to-in vivo extrapolation [18]. Furthermore, the obtained results are compared with clinical data. Finally, using liquid chromatography–high-resolution mass spectrometry (LC-HRMS), KM-819 metabolites are identified in rat, dog, and human hepatocytes and their metabolic pathways are proposed.

## 2. Results and Discussion

### 2.1. LC-MS/MS Method Development

**Figure 1:**
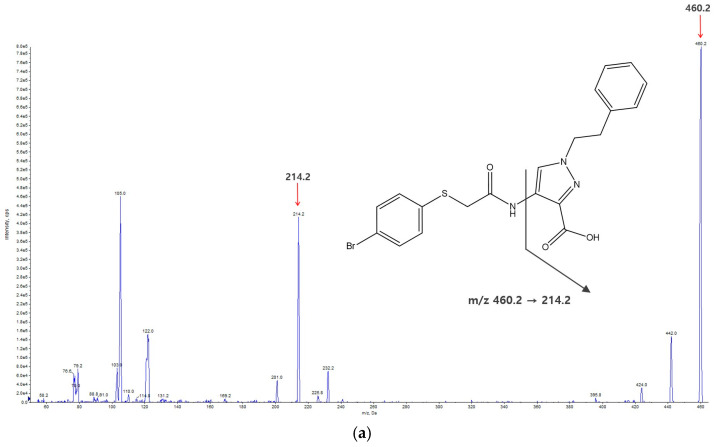

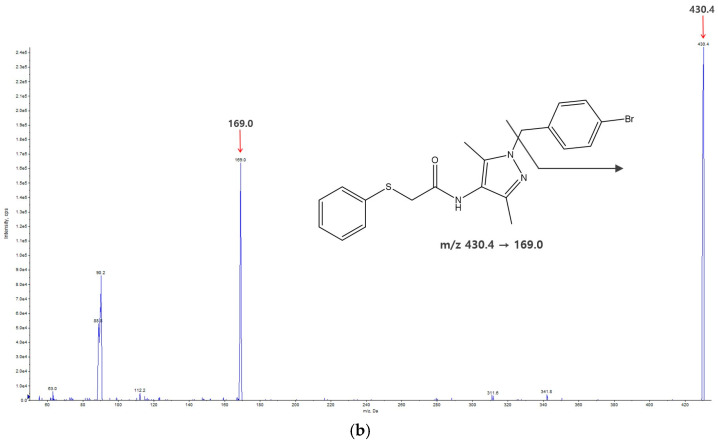
Product ion mass spectra of (**a**) KM-819 and (**b**) the internal standard (IS).

The precursor ions of KM-819 and the internal standard (IS) were scanned for detection in both the positive and negative electrospray ionization (ESI) modes. KM-819 and the IS produced protonated precursor ions [M + H]^+^ with mass-to-charge ratio (*m*/*z*) values of 460.2 and 430.4 in the positive ESI mode during the Q1 scan, respectively, and the signal intensities were superior to those obtained in the negative mode. The product ion transitions of KM-819 were detected at *m*/*z* 460.2 → 214.2 and 460.2 → 105.2; the transition at 460.2 → 214.2 was selected because it presented a superior sensitivity and linearity. In the case of the IS, the *m*/*z* 430.4 → 169.0 transition was selected. The mass spectra and structures of KM-819 and the IS are shown in Figure 1.

The column and mobile phase conditions were optimized to enhance the sensitivity and selectivity of KM-819 detection in rat plasma. The C18 column is most commonly used as a reversed-phase column for the analysis of nonpolar and small molecular compounds. The Zorbax Eclipse XDB-C18 (Agilent, Santa Clara, CA, USA) column was found to be suitable for analyzing KM-819 because of its long column life, good peak shape, and low baseline value. In the positive ESI mode, ionization is generally promoted under weakly acidic conditions; therefore, a mobile phase of water/acetonitrile containing 0.1% formic acid (FA) was used to increase the sensitivity for KM-819 detection.

### 2.2. LC-MS/MS Method Validation

**Figure 2:**
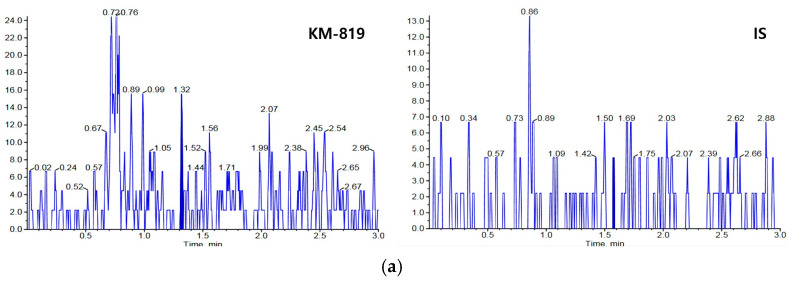

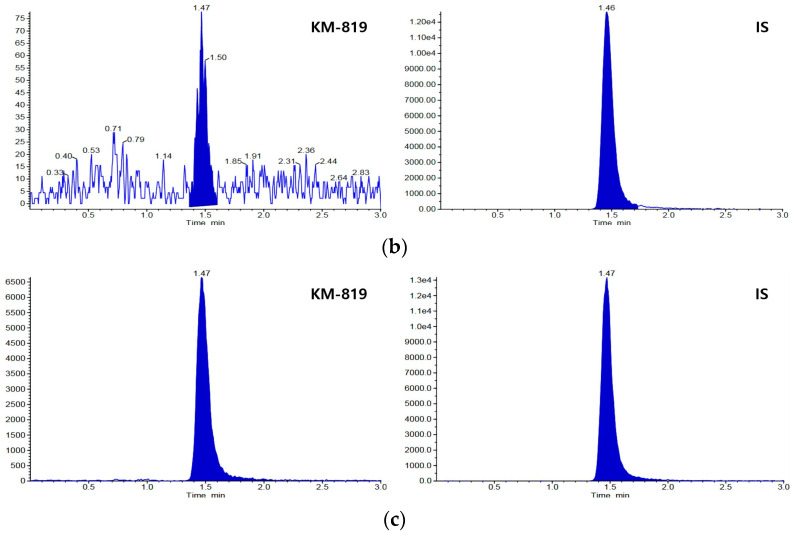
Chromatograms of KM-819 and the IS in rat plasma. (**a**) Blank rat plasma without KM-819 or the IS; (**b**) rat plasma spiked with 0.002 μg/mL (lower limit of quantification; LLOQ) KM-819 and 0.1 μg/mL IS; (**c**) plasma sample 1 h after intravenous injection of 0.5 mg/kg KM-819 to male Sprague-Dawley (SD) rats.

In rat plasma, the lower limit of quantification (LLOQ) was determined to be 0.002 μg/mL, which was more than five times higher than that of the blank samples. The retention times of KM-819 and IS were both 1.47 min and there was no interference peak at the retention time of KM-819 and IS in six individual blank rat plasma. The chromatograms of the blank plasma sample (no analyte or IS), the LLOQ sample (0.002 μg/mL), and the plasma samples obtained after intravenous injection of 0.5 mg/kg KM-819 to rats are presented in Figure 2. It was found that the retention times of KM-819 and the IS were constant during repeated analysis of the rat plasma samples and reproducible chromatograms free from interference were obtained. In addition, the carryover effect for KM-819 was negligible.

The calibration curve of KM-819 was plotted using nine calibration standards (0.002–10 μg/mL) and the analysis was repeated five times. KM-819 showed a good linearity in the rat plasma, with a correlation coefficient of r = 0.9942. The linear regression equation for the calibration curve was determined to be y = 0.00241x + 0.00054 using a weighting factor of 1/x^2^. To assess the suitability of this regression model, it was compared with a non-linear regression model and quadratic model using an F-test [19,20]. The results showed that the *p* value was greater than 0.05, indicating that the former was considered a more appropriate model. The precision and accuracy of the calibration standard were 4.3–11.2 and −8.4–6.7%, respectively, and all values were acceptable.

The precision and accuracy results for the intra- and inter-day analyses of the LLOQ and three quality control (QC) samples are summarized in Table 1. As indicated, the intra-day precision and accuracy ranged from 4.3 to 11.0 and from −4.8 to 3.8%, respectively, while the inter-day precision and accuracy ranged from 6.8 to 10.9 and from −5.9 to 7.3%, respectively, at the four concentrations of KM-819 examined herein. All data met the acceptance criteria of the FDA and EMA guidelines and verified that the bioanalytical method for KM-819 was reproducible, precise, and accurate. 

The matrix effect, recovery, and process efficiency of KM-819 and the IS in the rat plasma were then estimated from the low QC (LQC; 0.0045 μg/mL), middle QC (MQC; 0.45 μg/mL), and high QC (HQC; 9 μg/mL) samples. The matrix effects of KM-819 and the IS were 87.9 ± 5.1 and 93.0%, respectively, suggesting that KM-819 was not subjected to ion suppression or ion enhancement by the plasma matrix during LC-MS/MS analysis. In addition, the recoveries of KM-819 and IS were 91.9 ± 11.4 and 86.1%, respectively, and the process efficiencies of KM-819 and IS were 83.1 ± 2.2 and 80.1%, respectively. These results show that the pretreatment procedure is well established and that protein precipitation is an efficient pretreatment method for extracting KM-819 and the IS from rat plasma.

The dilution integrity was tested using 5-fold and 10-fold higher concentrations of the HQC (45 and 90 μg/mL, respectively). In the 5-fold and 10-fold diluted plasma samples, the coefficient of variation (CV, %) was found to be 1.9 and 0.7%, respectively, whereas the relative error (RE, %) was −6.2 and −7.5%, respectively, indicating that the dilution effect was insignificant.

The stability of KM-819 in the rat plasma under various storage and processing conditions (i.e., room temperature for 6 h, 4 weeks at −20 °C, 3 freeze–thaw cycles, and storage of the pretreated samples on the autosampler at 10 °C for 24 h) are listed in Table 2. The obtained results showed that in rat plasma, KM-819 satisfied the RE within ±15% under all conditions and was stable during sample processing and storage.

### 2.3. In-Vivo Pharmacokinetic Studies in Rats

**Figure 3:**
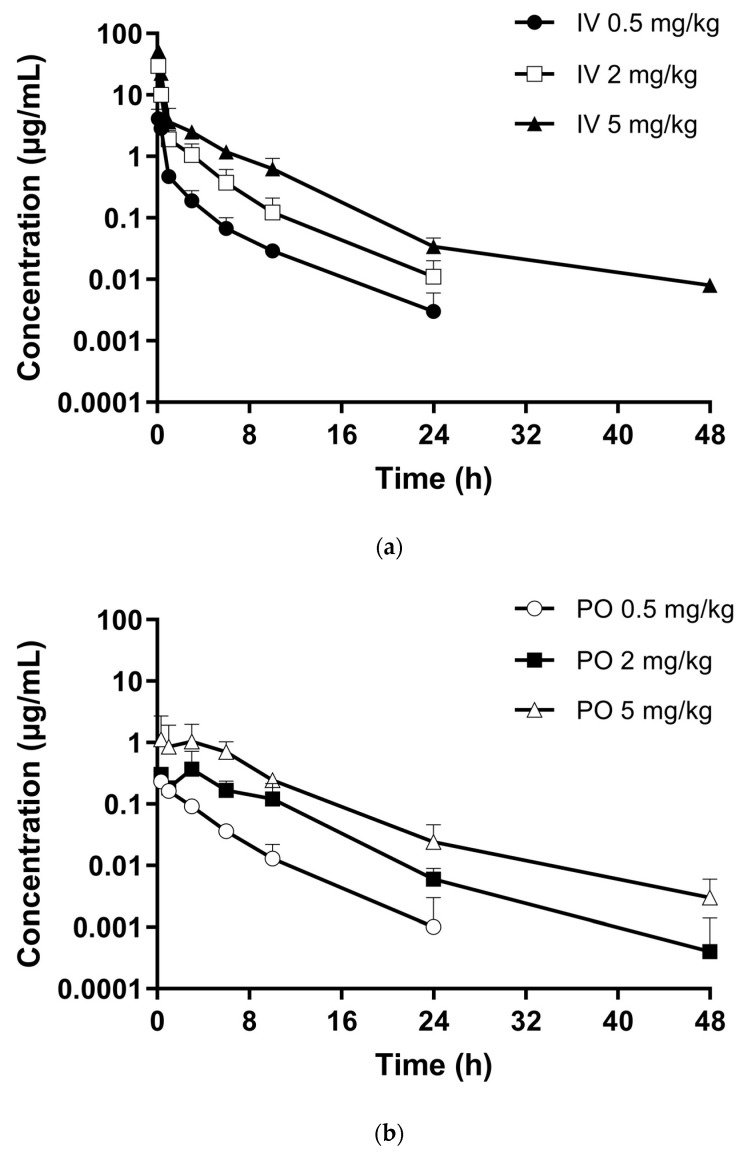
Plasma concentration–time curves of KM-819 after (**a**) intravenous (0.5 mg/kg, ●; 2 mg/kg, □; and 5 mg/kg, ▲) and (**b**) oral (0.5 mg/kg, ○; 2 mg/kg, ■; and 5 mg/kg, △) administration to fasted male SD rats. Data represent the mean ± standard deviation (*n* = 3; *n* = 4 for PO 5 mg/kg).

The developed LC-MS/MS method was subsequently applied to the pharmacokinetic study of KM-819 in rodents. The plasma concentration–time profiles recorded for KM-819 following intravenous (IV) or oral (PO) administration at doses of 0.5, 2, and 5 mg/kg in male rats are shown in Figure 3. The corresponding pharmacokinetic parameters are listed in Table 3.

After IV injection of KM-819 into rats at concentrations of 0.5, 2, and 5 mg/kg, the maximum plasma concentration (C_max_) values were determined to be 4.29 ± 1.71, 29.63 ± 3.60, and 52.33 ± 4.65 μg/mL, respectively, at 0.083 h, while the area under the plasma concentration versus time curve from 0 to the last measured concentration (AUC_last_) values were 3.76 ± 1.27, 18.97 ± 5.91, and 43.60 ± 1.37 μg·h/mL, respectively. The obtained AUC_last_ values showed a dose-dependent increase in the dose range of 0.5–5 mg/kg. In addition, the clearances (CL) were 142.8 ± 55.9, 113.8 ± 42.0, and 114.5 ± 3.6 mL/h/kg at 0.5, 2, and 5 mg/kg, respectively, which are lower values than that of the rat liver blood flow rate (3300 mL/h/kg [21]). This indicated that KM-819 is metabolically stable in rats. Furthermore, at doses of 0.5, 2, and 5 mg/kg, the volume of distributions at the steady state (V_ss_) for KM-819 in rats were determined to be 289.0 ± 118.7, 174.7 ± 16.8, and 351.6 ± 83.2 mL/kg, respectively. These values were lower than the extracellular fluid volume (0.4 L/kg), suggesting limited tissue distribution owing to the high plasma protein binding of KM-819 (99.96% in rats, 99.79% in dogs, and 99.96% in humans; unpublished data). Moreover, the half-lives (T_1/2_) at KM-819 doses of 0.5, 2, and 5 mg/kg were 4.11 ± 0.99, 3.54 ± 0.28, and 6.58 ± 1.02 h, respectively. Analysis of the dose correlation among the pharmacokinetic parameters using one-way analysis of variance (ANOVA) revealed no significant differences in the dose-normalized AUC_last_ (AUC_last_/D), CL, V_ss_, and mean residence time (MRT) values after intravenous injection at concentrations of 0.5, 2, and 5 mg/kg. It was therefore clear that KM-819 exhibited dose-independent pharmacokinetic properties at an intravenous dose range of 0.5–5 mg/kg in rats. However, the half-life differed significantly depending on the dosage. This was because the plasma concentration of KM-819 at 48 h was lower than the LLOQ. Therefore, an appropriate terminal elimination phase for T_1/2_ estimation was not obtained.

In terms of oral administration, the C_max_ values were 0.23 ± 0.07 μg/mL at 0.333 h, 0.44 ± 0.29 μg/mL at 3.111 h, and 1.71 ± 1.26 μg/mL at 3.833 h for doses of 0.5, 2, and 5 mg/kg, respectively. In addition, at these same doses, the AUC_last_ values were 0.76 ± 0.13, 3.07 ± 1.04, and 9.39 ± 4.50 μg·h/mL, respectively, indicating a dose-proportional increase over a dose range of 0.5–5 mg/kg. Furthermore, at doses of 0.5, 2, and 5 mg/kg, the T_1/2_ values were 3.43 ± 2.03, 3.92 ± 1.14, and 5.79 ± 2.36 h, respectively, and the MRT values were 4.06 ± 1.21, 6.46 ± 1.62, and 7.02 ± 2.50 h, respectively, indicating that the absorption rate slowed down due to absorption saturation as the dose increased. It was also found that the oral bioavailability (BA) was moderate in rats (i.e., 21.02 ± 2.96, 16.24 ± 5.50, and 21.62 ± 10.26%) over the 0.5–5 mg/kg dose range. Considering the low CL values, it implies a limitation in absorption rather than an impact from the first-pass effect in the liver. However, to understand this result, additional studies related to absorption, such as permeability assays (i.e., caco-2 cells permeability or parallel artificial membrane permeability assay), are needed. One-way ANOVA revealed no significant differences in the AUC_last_/D, T_1/2_, MRT, and BA values after oral administration of KM-819 at concentrations of 0.5, 2, and 5 mg/kg. These results suggest that in rats, KM-819 showed dose-independent pharmacokinetic properties at an oral dose range of 0.5–5 mg/kg.

### 2.4. Metabolic Stability in Hepatocytes

**Figure 4:**
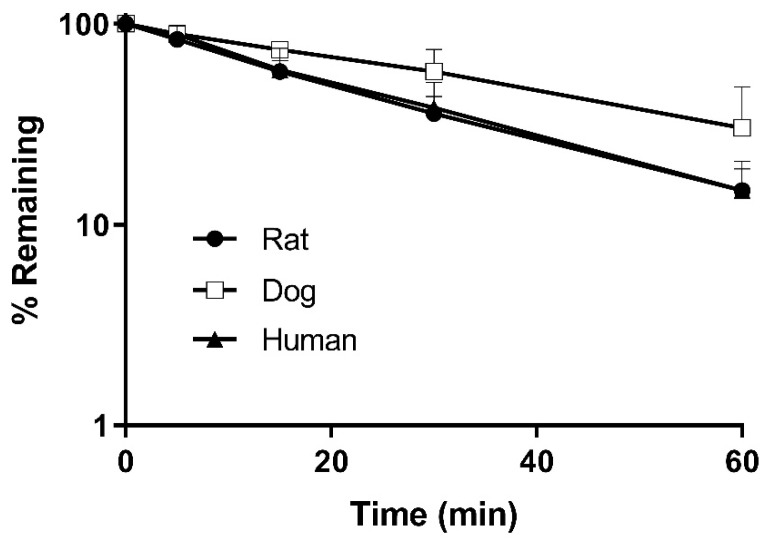
Residual amount of KM-819 (%) in (●) rat, (□) dog, and (▲) human hepatocytes. Data represent the mean ± standard deviation (*n* = 5).

The profiles of the mean remaining percentage of KM-819 versus time are presented in Figure 4 for rat, dog, and human hepatocytes. It was found that after incubating KM-819 (1 μM) with rat, dog, and human hepatocytes for 60 min, the amounts of unconverted KM-819 were 14.8, 30.2, and 14.8%, respectively. In addition, the half-lives were 21.8, 35.3, and 21.7 min in the rat, dog, and human hepatocytes, respectively, indicating moderate stabilities; it was clear that the half-life decreased in larger species.

Table 4 summarizes the in vitro T_1/2_, unbound hepatic intrinsic clearance (CL_u,int_), and hepatic clearance (CL_H_) values calculated from the metabolic stability results. As shown, the estimated CL_H_ values were 15.9, 57.7, and 8.0 mL/h/kg in rats, dogs, and humans, respectively. A scaling factor of 7.8 was determined by comparing the in vitro CL_H_ values to the mean CL values (i.e., 113.8–142.8 mL/h/kg) obtained from the in vivo pharmacokinetic studies in rats. Applying a scaling factor to the in vitro CL_H_ in humans, the human clearance was predicted to be 61.8 mL/h/kg. Compared to the CL value from a phase 1 clinical trial of KM-819 in healthy people (83.6–155.1 mL/h/kg), the predicted CL value was slightly lower but it is noteworthy that it was within twice the average value [13]. Furthermore, the significant difference between the in vitro CL_H_ and in vivo CL values shows that hepatic metabolism may not be the major elimination route of KM-819. Therefore, further studies such as extrahepatic metabolism or excretion are needed to elucidate this difference. Moreover, in previous microsomal stability tests, the half-life could not be determined because KM-819 was extremely stable for 60 min in rat, dog, and human microsomes (unpublished data). Considering the hepatocyte and microsomal stability results, phase 2 metabolism appeared to be dominant over phase 1 metabolism. This result is consistent with the metabolic identification study discussed later.

### 2.5. Metabolic Identification of KM-819

**Figure 5:**
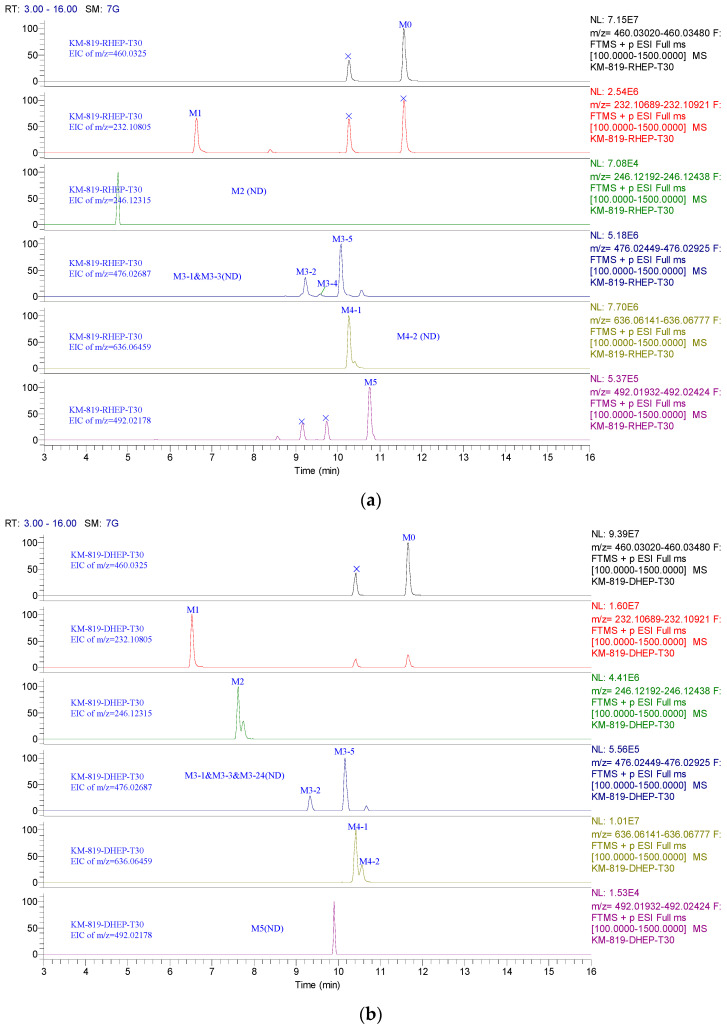

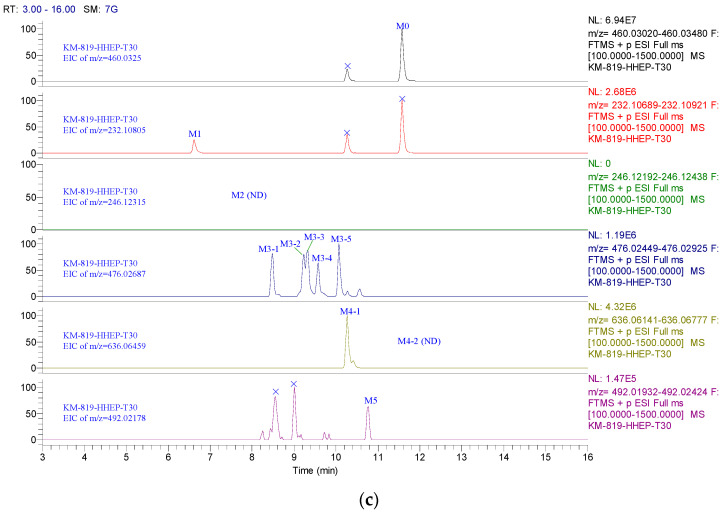
Liquid chromatography–high resolution mass spectrometry extracted ion chromatogram (LC–HRMS EIC) of KM-819 and its metabolites incubated with (**a**) rat, (**b**) dog, and (**c**) human hepatocytes.

Subsequently, the KM-819 metabolites were profiled in rat, dog, and human hepatocytes using a Q Exactive HF-X Hybrid Quadrupole-Orbitrap mass spectrometer. Figure 5 gives the liquid chromatography–high-resolution mass spectrometry (LC-HRMS) extracted ion chromatogram (EIC) of KM-819, showing the metabolites obtained from hepatocytes after incubation for 30 min. It was found that KM-819 was transformed into 10 potential metabolites, which were tentatively identified based on their mass shifts, fragment ions, and retention times (Table 5) [22]. The MS/MS spectra of the parent drug and individual metabolites are provided in the Supplementary Materials.

More specifically, it was found that metabolite M1 possessed a protonated ion at *m*/*z* 232.10806, which was identified as a dealkylated product, indicating the loss of C_8_H_5_OBrS from the parent drug. The protonated ion of M2 was observed at *m*/*z* 246.12350 and the loss of C_7_H_3_OBrS from the parent drug corresponds to a dealkylated and methylated metabolite of KM-819. The metabolites M3-1, M3-2, M3-3, M3-4, and M3-5 possessed *m*/*z* values of 476.02795, 476.02734, 476.02805, 476.02682, and 476.02792, respectively, and were identified as mono-oxidation products with molecular weights approximately 16 Da higher than that of the parent drug. Furthermore, M4-1 and M4-2 had *m*/*z* values of 636.06372 and 636.06268, respectively, and were suggested to be glucuronide-conjugated metabolites with molecular weights approximately 176 Da higher than that of KM-819. The protonated ion of M5 had an *m*/*z* of 492.02161 and was identified as the parent compound with two oxygen atoms added, thereby corresponding to a di-oxidation metabolite of KM-819.

The metabolites of KM-819 in the hepatocytes are species-dependent. For example, in rat hepatocytes, six metabolites (M1, M3-2, M3-4, M3-5, M4-1, and M5) were detected using LC-HRMS (Figure 5a). Based on the relative ultraviolet (UV) abundance (%), KM-819 was predominantly present as the parent drug (63.67%), whereas the most abundant metabolites were M4-1 (31.97%) and M3-5 (4.36%). Specific metabolites were not observed in the rat hepatocytes. In the dog hepatocytes, the parent drug was extensively metabolized and six metabolites (M1, M2, M3-2, M3-5, M4-1, and M4-2) were detected using LC-HRMS (Figure 5b). M4-1 and M4-2 were the major metabolites (49.95 and 4.20%, respectively) based on their normalized UV peak areas, wherein the parent drug was still present with an abundance of 45.85%. M2 was identified as a specific metabolite in dog hepatocytes. In human hepatocytes, the parent drug was found to be stable (relative UV abundance of 73.76%) and eight metabolites (M1, M3-1, M3-2, M3-3, M3-4, M3-5, M4-1, and M5) were detected by LC-HRMS (Figure 5c); M3-1 and M3-3 were identified as human-specific. The most abundant metabolite was M4-1 (21.19%) and the minor metabolites were M3-3 (2.59%) and M3-4 (2.45%).

**Figure 6:**
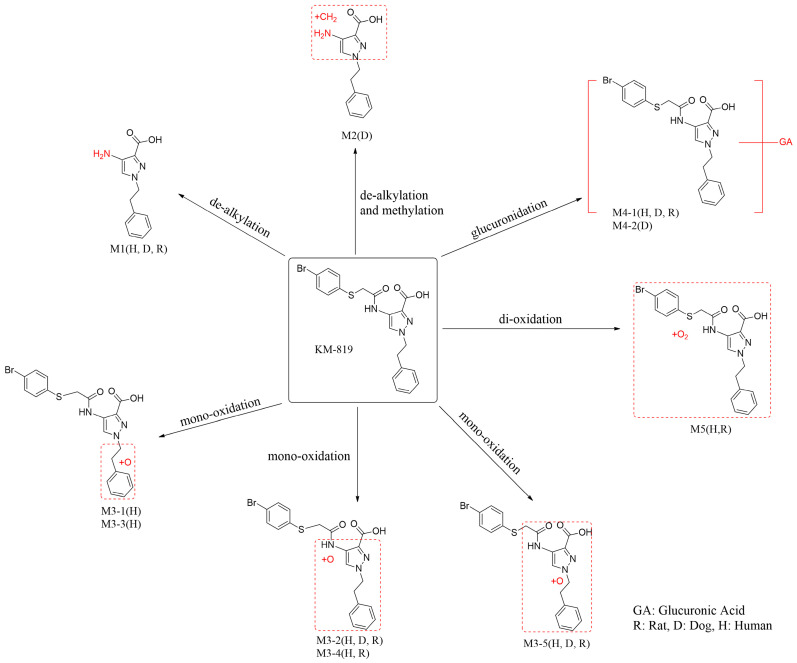
Proposed metabolic pathways for KM-819 in rat, dog, and human hepatocytes.

Finally, the metabolic pathways of KM-819 in the rat, dog, and human hepatocytes are shown in Figure 6. As indicated, KM-819 remained mostly unchanged in the hepatocytes and its major metabolic pathways included glucuronide conjugation, mono-oxidation, and dealkylation. Because M3-1 and M3-3 are human-specific metabolites that have not been detected in rats and dogs, it is necessary to confirm whether they are detected in plasma in clinical trials and to identify their metabolites in other species, such as monkeys. However, it is unlikely that M3-1 and M3-3 account for more than 10% of the total drug-related exposure because the production of M3-1 and M3-3 was low and about 1/10 of the main metabolite is the glucuronide-binding form. These results suggest that the need for additional toxicity tests is low [23]. However, because these metabolites were investigated in vitro, it has limitations in perfectly mimicking the actual physiological conditions. Furthermore, considering that hepatic metabolism is not the major metabolic route for KM-819, an in vivo system may be more appropriate than an in vitro system. Therefore, in vivo metabolites should be identified and compared with in vitro metabolites and subsequent metabolite studies should be considered. Overall, rats produced human-like metabolites but dogs did not, indicating that rats have a metabolic profile similar to that of humans. Therefore, rats are considered a more suitable animal species for predicting the human pharmacokinetic profile of KM-819.

## 3. Materials and Methods

### 3.1. Materials

KM-819 (Lot No. 55816001) was kindly provided from Kainos Medicine (Seongnam, Korea). *N*-[1-(4-Bromobenzyl)-3,5-dimethyl-1*H*-pyrazol-4-yl]-2-(phenylsulfanyl)acetamide (cat. no. MCULE-6381883653), the IS, was purchased from Mcule Inc. (Palo Alto, CA, USA). High-performance liquid chromatography (HPLC)-grade acetonitrile (cat. no. UN1648), distilled water (cat. no. 4218-88), and methanol (cat. no. AH230-4) were obtained from J.T. Baker (Phillipsburg, NJ, USA). Plasma was prepared from heparinized male Sprague-Dawley (SD) rats in our laboratory. Rat (cat. no. HEP134; Biopredic international, Rennes, France), dog (cat. no. BQD1000.H15B; Biopredic international, Rennes, France), and human (cat. no. SXTHPCH10; XenoTech, Kansas City, MO, USA) hepatocytes were used in this study. Dimethyl sulfoxide (DMSO; cat. no. D2660, Sigma-Aldrich, St. Louis, MO, USA), a 1M sodium hydroxide solution (NaOH; cat. no. 7576-3700, Daejung Chemicals, Siheung, Republic of Korea), 98% formic acid (FA; cat. no. 16233-73, Kanto Chemical, Tokyo, Japan), and polyethylene glycol 400 (PEG 400; cat. no. P0638; Samchun Chemicals, Pyeongtaek, Republic of Korea) were also used in this study. All chemicals and materials were of analytical grade or higher.

### 3.2. Analytical Methods

The concentration of KM-819 in the plasma was analyzed using a combination of an Agilent 1100 HPLC system (Agilent Technologies, Santa Clara, CA, USA) and an API 4000 QTRAP (AB Sciex, Framingham, MA, USA) triple quadrupole mass spectrometer. A Zorbax Eclipse XDB-C18 (5 µm, 2.1 × 50 mm, Agilent, Santa Clara, CA, USA) column connected to a Zorbax Eclipse XDB-C18 guard column (5 µm, 2.1 × 12.5 mm, Agilent, Santa Clara, CA, USA) was used as the reverse phase column. The mobile phase consisted of a mixture of 0.1% FA in distilled water and 0.1% FA in acetonitrile (30:70, *v*/*v*). Separation was achieved using isocratic elution at a flow rate of 0.3 mL/min with an injection volume of 5 µL. During analysis, the column oven and autosampler chamber were maintained at 40 and 10 °C, respectively. The MS/MS system was operated in the positive ionization mode at the ESI interface. Ion detection was performed in the multiple reaction mode (MRM) and the mass-to-charge ratios (*m*/*z*) were 460.2 → 214.2 and 430.4 → 169.0 for KM-819 and the IS, respectively. The optimized MS parameters were as follows: curtain gas pressure, 20 psi; source temperature, 600 °C; ion voltage, 5500 V; nebulizer gas pressure, 50 psi; turbo gas pressure, 50 psi; entrance potential (EP), 10 V; declustering potential (DP), 71 and 96 V; collision energy (CE), 35 V; and collision cell exit potential (CXP), 36 and 28 V for KM-819 and the IS, respectively. The peak areas were integrated automatically using Analyst software version 1.6.4 (Applied Biosystems/MDS SCIEX, Framingham, MA, USA).

In vitro metabolite studies of KM-819 in the hepatocytes were performed using high-performance liquid chromatography–ultraviolet–high-resolution mass spectrometry (HPLC-UV-HRMS). All samples were quenched with acetonitrile and analyzed using a Q Exactive HF-X Hybrid Quadrupole-Orbitrap mass spectrometer (Thermo Scientific, Waltham, MA, USA). A Waters ACQUITY UPLC BEH C18 column (1.7 µm, 2.1 × 100 mm, Waters, Milford, MA, USA) was used and the column chamber was maintained at 40 °C. The mobile phase consisted of 0.1% FA in distilled water (A) and 0.1% FA in acetonitrile (B), with gradient elution as follows: 0–3 min, 2% B; 3–12 min, 10–75% B; 12.01–15 min, 75–95% B; 15.01–18 min, 95–98% B, and 18.01–20 min, 2% B. The flow rate was 0.3 mL/min and samples were analyzed at an ultraviolet wavelength of 190–400 nm. The MS/MS spectra of KM-819 and its metabolites were obtained using the positive-ion electrospray mode. The source conditions were optimized as follows: spray voltage, 3.5 kV; aux gas heater temperature, 350 °C; capillary temperature, 320 °C; sheath gas flow rate, 40 L/h; aux gas flow rate, 15 L/h; sweep gas flow rate, 1 L/h.

### 3.3. Preparation of the Standard, Quality Control, and Plasma Samples

A KM-819 stock solution was prepared at a concentration of 1 mg/mL in methanol. Working standard solutions for the standard and QC samples were prepared independently. To prepare the calibration standard samples and the QC samples, KM-819 was serially diluted with acetonitrile to give the following 10-fold concentrated working standard solutions: 0.02, 0.05, 0.1, 0.3, 1, 3, 10, 30, and 100 μg/mL for the standard samples and 0.045, 4.5, and 90 μg/mL for the QC samples. After that, the 10-fold concentrated working solution (5 µL) was added to blank plasma (45 µL) and mixed and then IS (50 µL, 0.1 μg/mL in acetonitrile) and acetonitrile (150 µL) were added to induce protein precipitation. The mixture was vigorously mixed for 10 min and then subjected to centrifugation at 13,500 rpm for 10 min. Subsequently, an aliquot of the supernatant (150 μL) was taken for analysis and a volume of 5 μL was injected into the LC-MS/MS system.

Each rat plasma sample (50 µL) was spiked with the IS (50 µL, 0.1 μg/mL in acetonitrile) and acetonitrile (150 µL) to induce protein precipitation. After intravenous injection at doses of 2 or 5 mg/kg, samples from the initial two-time points exceeding the quantitative range were diluted 10-fold with blank plasma. The solution was vigorously mixed for 10 min and then subjected to centrifugation at 13,500 rpm for 10 min. Subsequently, an aliquot of the supernatant (150 μL) was taken for analysis and a volume of 5 μL was injected into the LC-MS/MS system.

### 3.4. Method Validation

The quantitative analysis of KM-819 in the rat plasma samples was fully validated in accordance with FDA and EMA guidelines [16,17]. The major validation parameters were specificity, linearity, precision and accuracy, matrix effects, recovery, process efficiency, dilution integrity, and stability.

The specificity was assessed using six different sets of blank rat plasma samples to demonstrate the absence of chromatographic interference from endogenous substances. The sensitivity was evaluated at the LLOQ, which was five times higher than the blank sample response.

Carryover was evaluated by analyzing blank samples immediately after injecting the upper limit of quantification (ULOQ). If the peak area of KM-819 in the blank sample did not exceed 20% of the LLOQ, the carryover was considered to be negligible.

The calibration curve was acquired by plotting the peak area ratios of KM-819 to the IS against the nominal concentrations of the calibration standards (i.e., 0.002, 0.005, 0.01, 0.03, 0.1, 0.3, 1, 3, and 10 μg/mL). To fit the calibration curve, least-squares linear regression with a 1/x^2^ weighting factor was applied. The linearity was validated using the correlation coefficient (r) and the acceptance criterion for linearity was r > 0.99.

The precision and accuracy were evaluated by analyzing the LLOQ (0.002 μg/mL), LQC (0.0045 μg/mL), MQC (0.45 μg/mL), and HQC (9 μg/mL). The intraday precision and accuracy were obtained by analyzing five replicates for each concentration per day and interday precision and accuracy were determined by analyzing the data for three consecutive days. The precision and accuracy were expressed as the CV (%) and RE (%), respectively. The acceptance criteria were within ±15%, with the exception of the LLOQ, which was within ±20%.

The matrix effect, recovery, and process efficiency were measured at three different QC concentrations. The matrix effect was determined as a percentage by dividing the peak areas observed for KM-819 dissolved in acetonitrile (Set 1) and KM-819 spiked into the blank plasma extract (Set 2). The recovery was calculated as a percentage by dividing the peak areas observed in the pretreated KM-819 (Set 3) and in Set 2. The process efficiency was estimated as a percentage by dividing the peak areas of Sets 3 and 1.

The dilution integrity was also investigated to assess whether sample dilution affected the analysis when the samples were diluted out of the quantification range. For this purpose, KM-819-spiked rat plasma samples at 45 and 90 μg/mL (5- and 10-fold HQC) were diluted 1/5 and 1/10 with blank rat plasma, respectively. The analysis was repeated 5 times for each dilution factor, with the targets being a CV of ±15% and a RE of ±15%.

The stability test was performed at both the LQC and the HQC to evaluate the stability of the plasma samples during handling and storage, according to the following conditions: 6 h at room temperature (RT), 4 weeks at −20 °C, three freeze–thaw cycles from −20 °C to RT, and storage of the pretreated sample in the autosampler at 10 °C for 24 h. The stability test analyzed five replicates, with the target being a RE of ±15% at each QC level.

### 3.5. In-Vivo Pharmacokinetic Studies in Rat

Specific pathogen-free (SPF) rats were used in pharmacokinetic studies. Nineteen healthy male Sprague-Dawley (SD) rats (7 weeks old, 200–220 g) were supplied by Orient Bio Inc. (Seongnam, Republic of Korea; License Number: 20090275184). The animals were stabilized for 1 week under a suitable temperature (20–25 °C), humidity (40–60%), and light–dark cycle (12 h) in the animal room. Water and food were freely available but only water was supplied for 15 h before the experiment and the subjects were fasted for 4 h after drug administration. This animal study was approved by the Institutional Animal Care and Use Committee of Chungnam National University (202203A-CNU-056; Daejeon, Republic of Korea).

For intravenous and oral administration, KM-819 was dissolved at concentrations of 0.5, 2, and 5 mg/mL in a vehicle containing 10% DMSO, 39.6% PEG400, 10% 1M NaOH, 0.8% acetic acid, and 39.6% distilled water. The KM-819 dosing solution was intravenously (IV) injected into the tail vein or orally (PO) administered to the rats using a gavage needle, with single doses of 0.5, 2, and 5 mg/kg being used in both the IV and PO groups.

In the case of time interval selection, at least two points were needed in the early period after injection to confirm early distribution, so two points were selected within 0.5 h (0.083 and 0.33 h). For the oral administration group, 5 min were excluded considering absorption. Furthermore, to determine the half-life, at least three time points are required in the terminal phase and it is recommended to include 3–4 points between the initial and final three points [24]. In rodent pharmacokinetic studies, the final time is commonly selected at 24 h. However, in this study, considering the potential for overestimation or underestimation of pharmacokinetic parameters with increasing doses, the final time point was extended to 48 h. Therefore, in this paper, the following time points were selected based on reference and experience: 0.083 (IV only), 0.33, 1, 3, 6, 10, 24, and 48 h after administration.

At times of 0.083 (IV only), 0.33, 1, 3, 6, 10, 24, and 48 h after administration, blood (150 μL) was collected from the jugular vein using a heparinized syringe. The collected blood samples were then centrifuged at 3000 rpm for 10 min and plasma (50 μL) was taken and stored frozen at −20 °C until required for LC-MS/MS analysis.

Pharmacokinetic parameters were calculated using non-compartmental analysis (NCA) in Phoenix^®^ 8.3 software (Certara L.P., Princeton, NJ, USA) as follows. The C_max_ and time to reach C_max_ (T_max_) were determined directly from the plasma concentration–time curve. The first-order elimination rate constant (k_e_) was determined from the slope of linear regression in the terminal phase of the log-linear plot. The T_1/2_ was obtained using the expression ln2/k_e_. The CL, V_ss_, and MRT were estimated using moment analysis. The area under the plasma concentration versus time curve from 0 to infinity (AUC_inf_) was calculated using the linear trapezoidal rule and the standard area extrapolation method [24].

### 3.6. Metabolic Stability in Hepatocytes

The metabolic stability of KM-819 was subsequently investigated in rat, dog, and human hepatocytes. Initially, KM-819 was dissolved in DMSO to prepare a 10 mM stock solution. Subsequently, the stock solution was diluted to 2 μM using William’s Medium E to prepare a 2-fold dosing solution. An aliquot (40 μL) of each hepatocyte suspension (2 × 10^6^ cells/mL) was added to 96-well plates at different time points. After spiking with an aliquot (40 μL) of the pre-warmed 2-fold dosing solution, it was incubated at 37 °C with 5% CO_2_ at 110 rpm. The reactions were terminated at the desired time points (0, 5, 15, 30, and 60 min) by adding ice-cold acetonitrile containing the IS (240 μL). The plate was then shaken for 2 min and subjected to centrifugation at 6000 rpm for 15 min. For analysis, an aliquot (100 μL) of the supernatant was mixed with an equal volume of distilled water and injected into the LC-MS/MS system [25].

The T_1/2_, k_e_, hepatocyte intrinsic clearance (CL_int,vitro_), CL_u,int_, and CL_H_ values were calculated using the metabolic stability results. The human clearance was predicted using an in vitro-to-in vivo extrapolation (IVIVE) and compared with clinical data [13,18,19,22].

### 3.7. Metabolic Identification of KM-819

The in vitro metabolites were investigated by incubating KM-819 with rat, dog, and human hepatocytes. The metabolites of KM-819 were identified by comparing the zero-time samples and the samples incubated for 30 min. An aliquot (100 μL) of KM-819 at a concentration of 20 μM was incubated with the rat, dog, or human hepatocyte suspensions (100 μL, 2 × 10^6^ cells/mL) at 37 °C for 30 min and the reaction was terminated by adding acetonitrile containing 0.1% FA (600 µL) to the reaction tube. The mixture was then vortexed for 5 min and subjected to centrifugation at 18,000 rpm for 10 min to remove the proteins. The supernatant (700 μL) was transferred to a clean tube and evaporated to dryness under a stream of N_2_ gas and the residue was reconstituted with a water/acetonitrile solution containing 0.1% FA (200 μL, 25:75, *v*/*v*). After vortexing for 5 min and further centrifugation at 18,000 rpm for 5 min, an aliquot (2 μL) of the supernatant was injected into the HPLC-UV-HRMS system for metabolite profiling and identification [23,26].

### 3.8. Statistics

The data are presented as the mean ± standard deviation for all groups. Pharmacokinetic parameters (AUC_last_/D, CL, V_ss_, MRT, and T_1/2_) for the three dosage groups were compared using one-way ANOVA in Prism 9.4 (GraphPad Software, San Diego, CA, USA). ANOVA revealed a statistically significant difference when the *p* value was <0.05.

## 4. Conclusions

In conclusion, a high-performance liquid chromatography–tandem mass spectrometry (HPLC-MS/MS) method was developed for the determination of KM-819 in rat plasma. The developed analytical method was validated as reliable and reproducible by satisfying the requirements of both the FDA and EMA. This sensitive, simple, and rapid method was successfully applied to rat pharmacokinetic studies. It was found that KM-819 exhibited dose-independent pharmacokinetics after intravenous and oral administration to rats at doses of 0.5–5 mg/kg. In addition, KM-819 exhibited moderate stabilities in rat, dog, and human hepatocytes. In the metabolite identification studies, six metabolites were observed in rat and dog hepatocytes and eight metabolites, including two human-specific metabolites, were observed in human hepatocytes. The major metabolic pathways involved were glucuronic acid conjugation and mono-oxidation. Overall, these findings contribute to a better understanding of the pharmacokinetic characteristics of KM-819, thereby aiding future clinical studies.

## Figures and Tables

**Table 1:** Intra- and inter-day precisions and accuracies for KM-819 analysis in rat plasma.

Nominal Concentration (μg/mL)	Measured Concentration (μg/mL)	Precision (CV, %)	Accuracy (RE, %)
Intra-day (*n* = 5)			
0.002	0.00218 ± 0.00023	11.0	3.8
0.0045	0.00453 ± 0.00022	4.9	0.8
0.45	0.458 ± 0.041	9.0	1.9
9	8.568 ± 0.368	4.3	−4.8
Inter-day (*n* = 15)			
0.002	0.00215 ± 0.00023	10.9	7.3
0.0045	0.00427 ± 0.00033	7.8	−5.1
0.45	0.463 ± 0.316	6.8	2.8
9	8.468 ± 0.658	7.8	−5.9

**Table 2:** Stability of KM-819 in rat plasma.

Storage Conditions	Nominal Concentration (μg/mL)	Stability (%)
6 h at room temperature (25 °C)	0.0045	101.3 ± 8.0
	9	102.7 ± 7.5
1 month at −20 °C	0.0045	97.7 ± 1.9
	9	100.1 ± 8.2
Freeze-thaw three-cycle	0.0045	93.9 ± 4.5
	9	99.1 ± 8.5
Processed sample in 10 °C autosampler for 24 h	0.0045	92.0 ± 5.7
	9	99.5 ± 10.4

Data presented as mean ± standard deviation (*n* = 5).

**Table 3:** Pharmacokinetic parameters of KM-819 after intravenous and oral administration to fasted male SD rats.

	IV			PO		
Dose (mg/kg)	0.5	2	5	0.5	2	5
T_max_ (h)	0.167 ± 0.140	0.083 ± 0.000	0.083 ± 0.000	0.333 ± 0.000	3.111 ± 2.835	3.833 ± 2.728
C_max_ (μg/mL)	4.29 ± 1.71	29.63 ± 3.60	52.33 ± 4.65	0.23 ± 0.07	0.44 ± 0.29	1.71 ± 1.26
T_1/2_ (h)	4.11 ± 0.99	3.54 ± 0.28	6.58 ± 1.02	3.43 ± 2.03	3.92 ± 1.14	5.79 ± 2.36
AUC_last_ (μg·h/mL)	3.76 ± 1.27	18.97 ± 5.91	43.60 ± 1.37	0.76 ± 0.13	3.07 ± 1.04	9.39 ± 4.50
AUC_inf_ (μg·h/mL)	3.82 ± 1.22	19.03 ± 5.96	43.68 ± 1.39	0.80 ± 0.11	3.09 ± 1.05	9.45 ± 4.48
AUC_last_/D	7.52 ± 2.55	9.49 ± 2.95	8.72 ± 0.27	1.51 ± 0.26	1.54 ± 0.52	1.88 ± 0.90
CL (mL/(h·kg))	142.8 ± 55.9	113.8 ± 42.0	114.5 ± 3.6	-	-	-
V_ss_ (mL/kg)	289.0 ± 118.7	174.7 ± 16.8	351.6 ± 83.2	-	-	-
MRT (h)	2.02 ± 0.10	1.69 ± 0.66	3.06 ± 0.67	4.06 ± 1.21	6.46 ± 1.62	7.02 ± 2.50
BA (%)	-	-	-	21.02 ± 2.96	16.24 ± 5.50	21.62 ± 10.26

IV, intravenous; PO, per oral; C_max_, maximum plasma concentration; T_max_, time to reach C_max_; T_1/2_, terminal elimination half-life; AUC, area under the plasma concentration–time curve; AUC/D, dose-normalized AUC; CL, systemic clearance; V_ss_, steady-state volume of distribution; MRT, mean residence time; BA, bioavailability. Data are presented as mean ± standard deviation (*n* = 3; *n* = 4 for PO 5 mg/kg); there are no significant differences among the PK parameters (*p* > 0.05).

**Table 4:** In vitro metabolic stability of KM-819 in rat, dog, and human hepatocytes.

Parameter	Rat	Dog	Human
T_1/2_ (min)	21.8	35.3	21.7
f_ub_	0.0004	0.0021	0.0004
CL_u,int_ (mL/h/kg)	39,027	28,238	19,546
CL_H_ (mL/h/kg)	15.9	57.7	8.0

T_1/2_, terminal elimination half-life; f_ub_, blood-unbound fraction of KM-819 (unpublished data); CL_u,int_, unbound hepatic intrinsic clearance; CL_H_, hepatic clearance.

**Table 5:** Summary of the metabolites of KM-819 in rat, dog, and human hepatocytes.

Peak ID	Formula	Mass Shift	*m*/*z*	Error (ppm)	Biotransformation	RT (min)	Species
Parent	C_20_H_18_N_3_O_3_BrS	-	460.03250	1.46	-	11.58	R, D, H
M1	C_12_H_13_N_3_O_2_	−227.92445	232.10805	0.04	de-alkylation	6.61	R, D, H
M2	C_13_H_15_N_3_O_2_	−213.90935	246.12315	−0.81	de-alkylation and methylation	7.62	D
M3-1	C_20_H_18_N_3_O_4_BrS	15.99437	476.02687	1.13	mono-oxidation	8.48	H
M3-2				−0.15		9.23	R, D, H
M3-3				1.32		9.31	H
M3-4				−1.24		9.57	R, H
M3-5				1.07		10.07	R, D, H
M4-1	C_26_H_26_N_3_O_9_BrS	176.03209	636.06459	−1.37	glucuronide conjugation	10.26	R, D, H
M4-2				−3.00		10.55	D
M5	C_20_H_18_N_3_O_5_BrS	31.98928	492.02178	−1.47	di-oxidation	10.77	R, H

*m*/*z*, mass-to-charge ratio; RT, retention time; R, rat; D, dog; H, human.

## Data Availability

The data presented in this study are available in the article.

## Supplementary Materials

The following supporting information can be downloaded at https://www.mdpi.com/article/10.3390/molecules29051004/s1. Figure S1: (a) MS/MS spectra of KM-819 and (b) putative fragmentation pathways of KM-819; Figure S2: MS/MS spectra of M1, the de-alkylation metabolite of KM-819, and structure elucidation; Figure S3: MS/MS spectra of M2, the De-alkylated and methylated metabolite of KM-819, and structure elucidation; Figure S4: MS/MS spectra of (a) M3-1, (b) M3-2, (c) M3-3, (d) M3-4, and (e) M3-5, the mono-oxidation metabolite of KM-819, and structure elucidation; Figure S5: MS/MS spectra of (a) M4-1 and (b) M4-2, the glucuronidation metabolite of KM-819, and structure elucidation; Figure S6: MS/MS spectra of M5, the di-oxidation metabolite of KM-819, and structure elucidation.
