# Supplementary material for: Rat Pharmacokinetics and In Vitro Metabolite Identification of KM-819, a Parkinson’s Disease Candidate, Using LC-MS/MS and LC-HRMS
Source: Molecules. 2024 Feb 25;29(5):1004. doi: 10.3390/molecules29051004 (PMC10934217; doi:10.3390/molecules29051004)

molecules

MDPI

Supplementary Materials

Rat Pharmacokinetics and In Vitro Metabolite Identification of KM-819, a Parkinson's Disease Candidate, Using LC-MS/MS and LC-HRMS

Hae-In Choi [1], Taeheon Kim [1], Jin Woo Kim [1], Gi Ju Lee [1], Jinyoung Choi [1], Yoon-Jee Chae [2], Eunhee Kim [3,4] and Tae-Sung Koo [1,*]

[1] Graduate School of New Drug Discovery and Development, Chungnam National University, Daejeon 34134, Republic of Korea; chi705@naver.com (H.-I.C.); thkim150@daewoong.co.kr (T.K.); dpslzk333@naver.com (J.W.K.); dlrlwn1234@naver.com (G.J.L.); jinyoung724@naver.com (J.C.)
[2] College of Pharmacy, Woosuk University, Wanju 55338, Republic of Korea; yjchae@woosuk.ac.kr
[3] College of Biological Sciences and Biotechnology, Chungnam National University, Daejeon 34134, Republic of Korea; eunhee@cnu.ac.kr
[4] Biopharmaceutical Division, Kainos Medicine Inc., Seongnam 13488, Republic of Korea
[*] Correspondence: kootae@cnu.ac.kr; Tel.: +82-42-821-8628

Supplementary Figure S1. (a) MS/MS spectra of KM-819 and (b) putative fragmentation pathways of KM-819

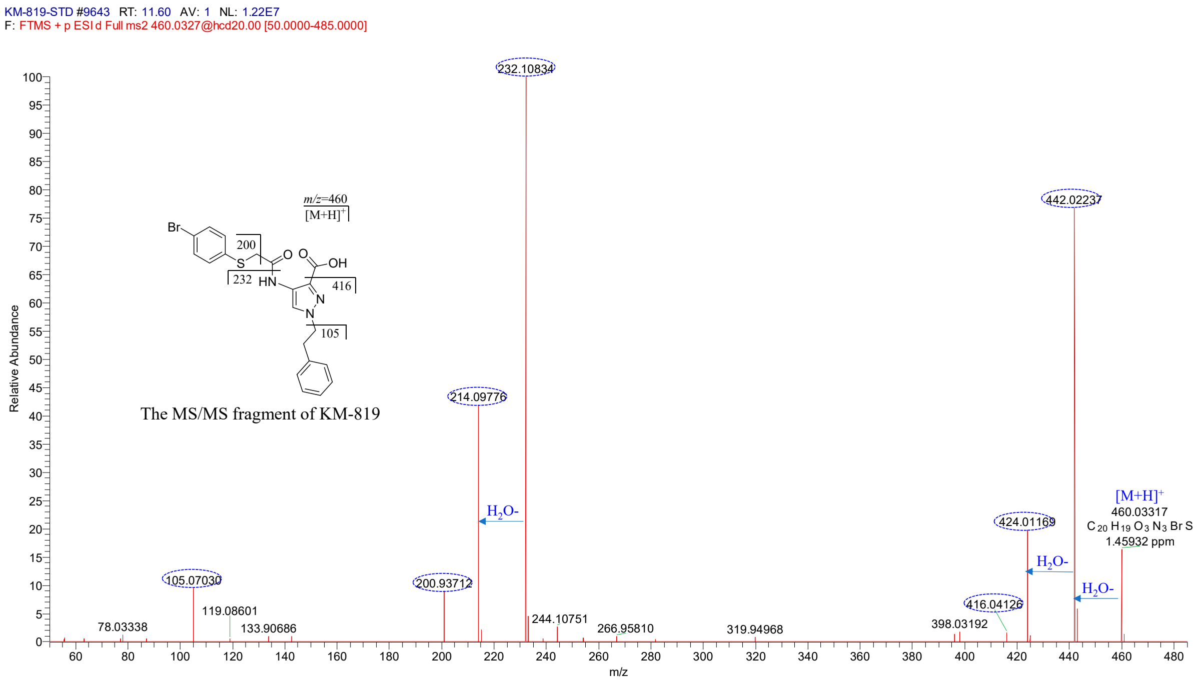

(**a**)

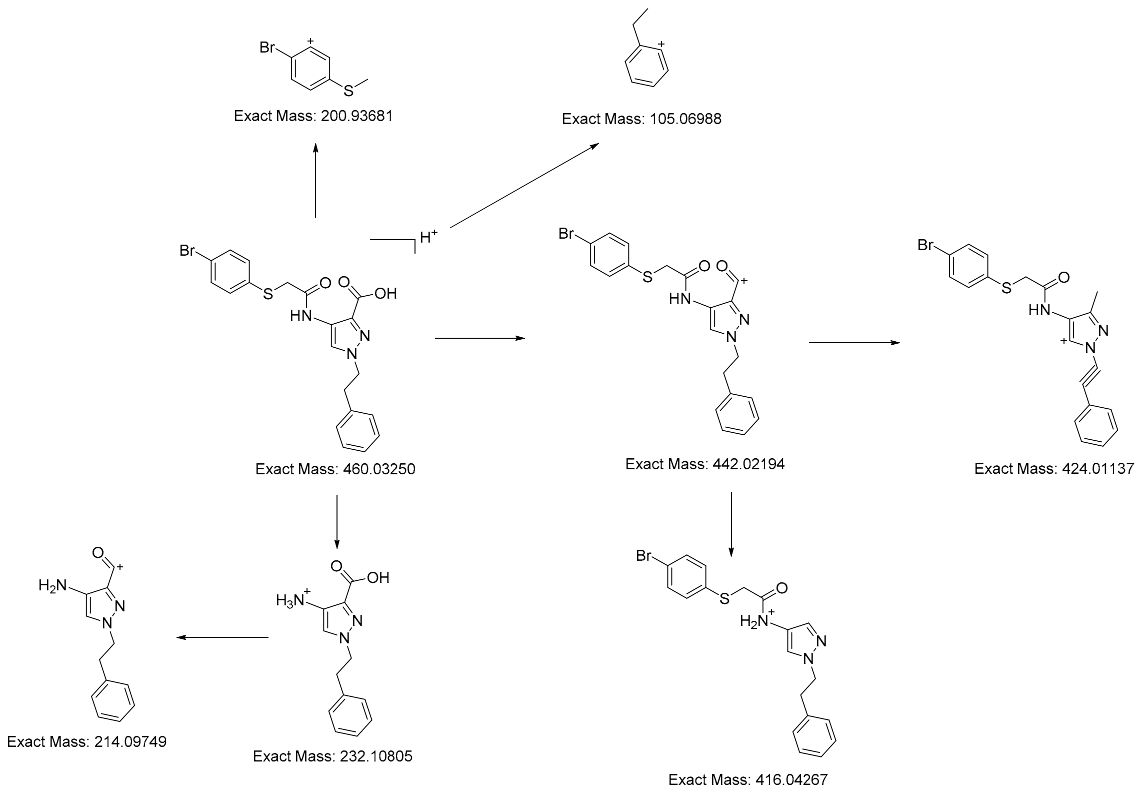

(**b**)

Supplementary Figure S2. MS/MS spectra of M1, the de-alkylation metabolite of KM-819, and structure elucidation

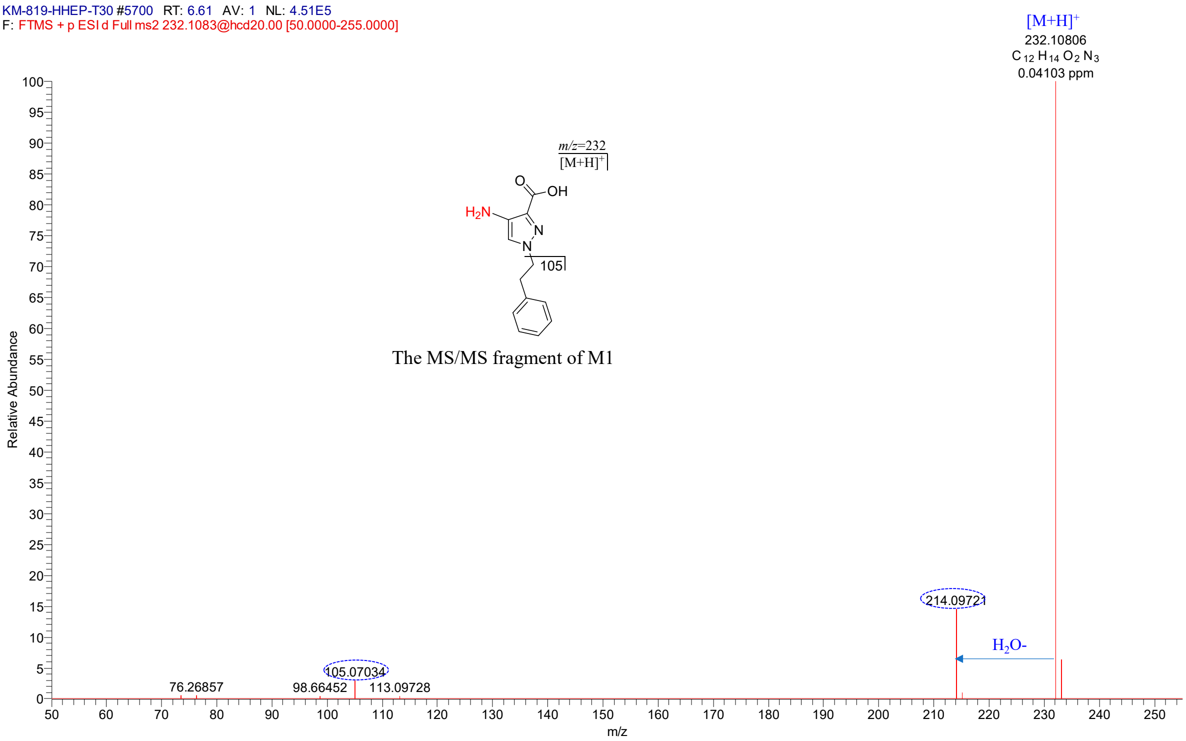

Supplementary Figure S3. MS/MS spectra of M2, the De-alkylated and methylated metabolite of KM-819, and structure elucidation

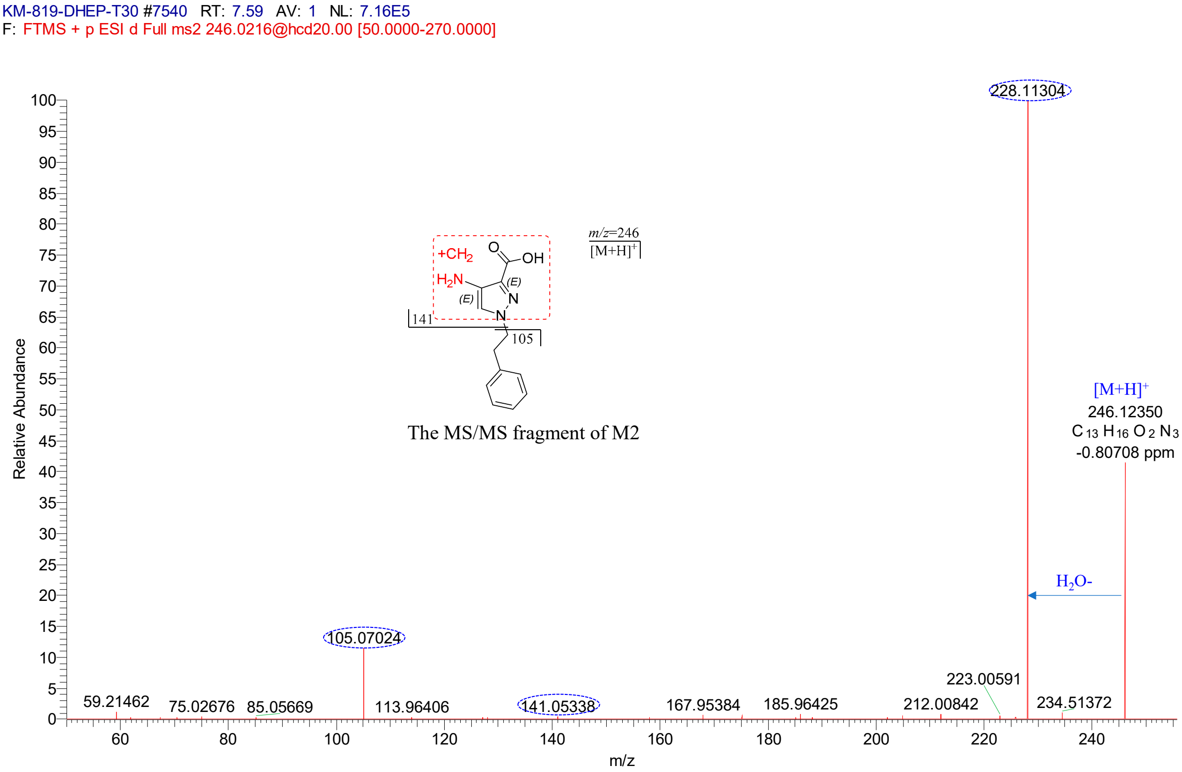

Supplementary Figure S4. MS/MS spectra of (a) M3-1, (b) M3-2, (c) M3-3, (d) M3-4, (e) M3-5, the mono-oxidation metabolite of KM-819, and structure elucidation

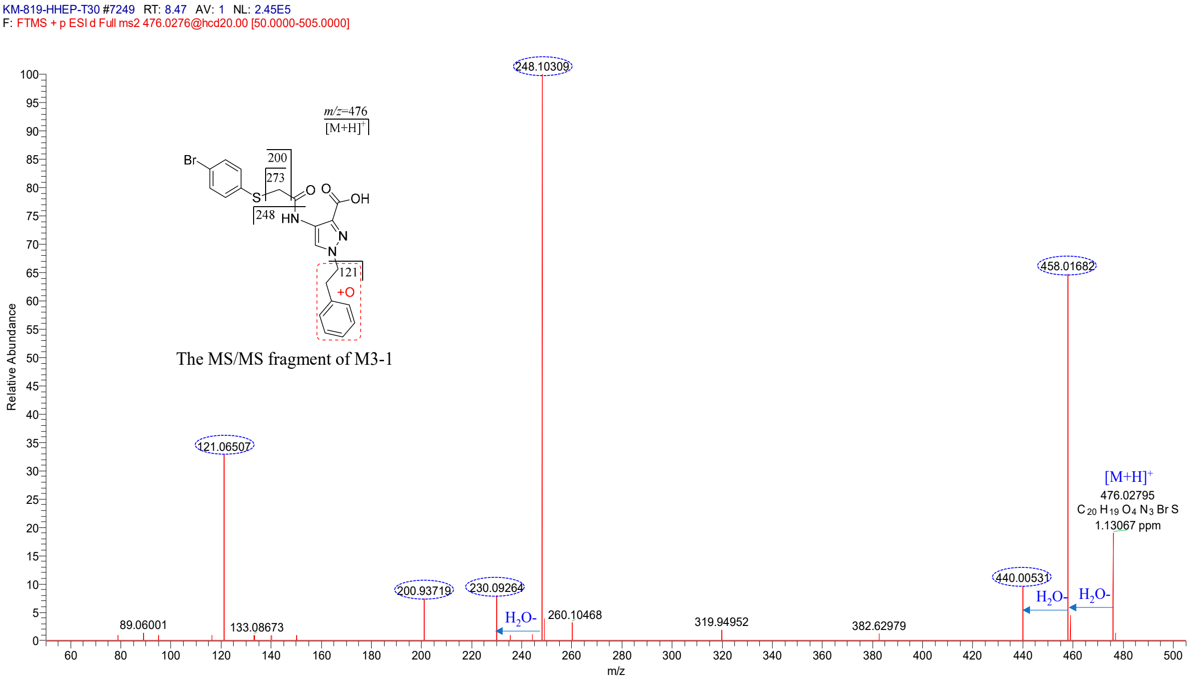

(**a**)

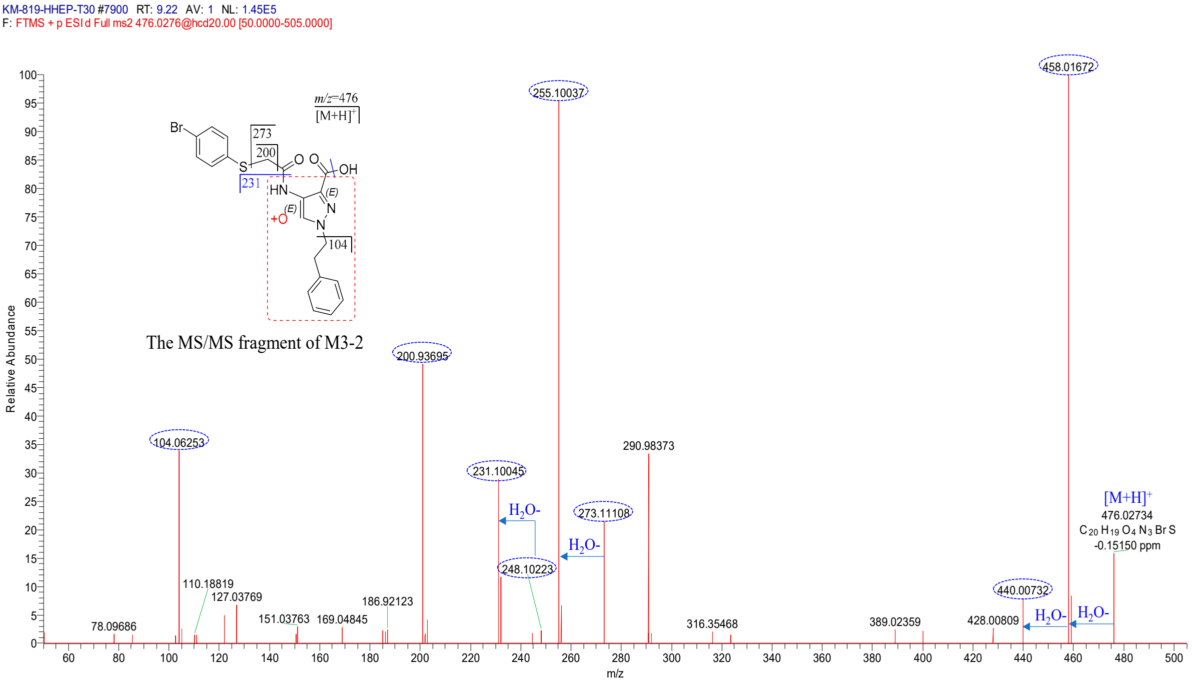

(**b**)

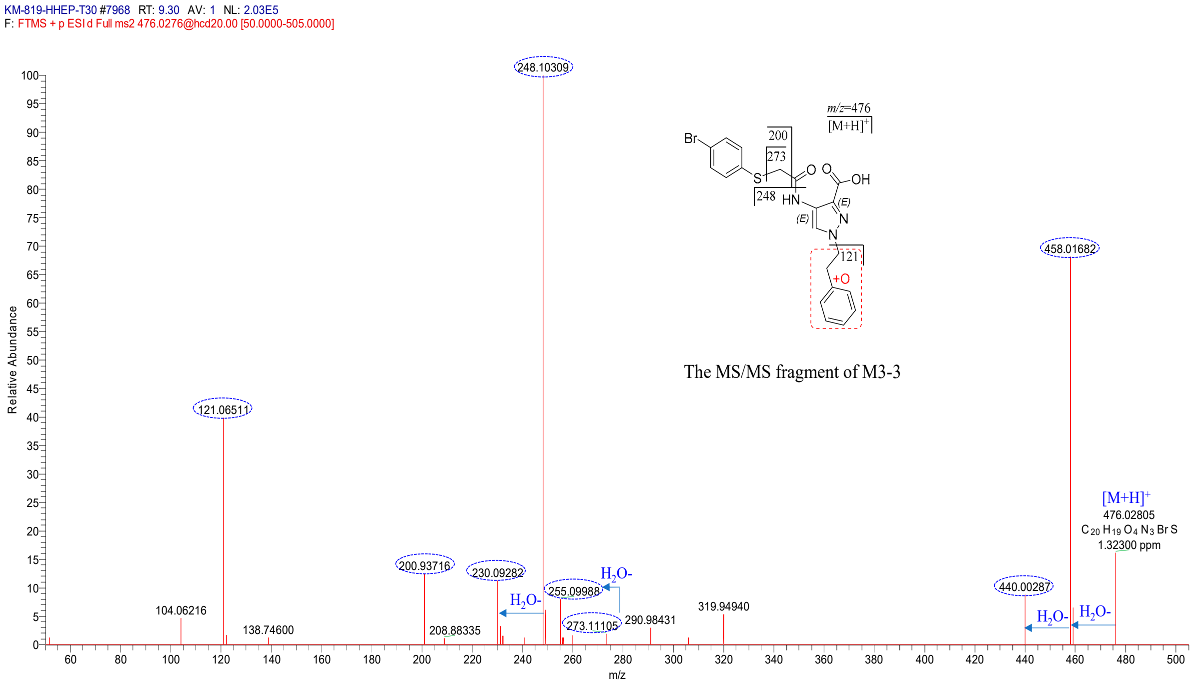

KM-819-HHEP-T30 #7968 RT: 9.30 AV: 1 NL: 2.03E5
F: FTMS + p ESI d Full ms2 476.0276@hcd20.00 [50.0000-505.0000]
Relative Abundance
m/z
248.10309
458.01682
121.06511
200.93716
230.09282
255.09988
273.11105
290.98431
319.94940
104.06216
138.74600
208.88335
440.00287
H2O-
[M+H]+
476.02805
C20 H19 O4 N3 Br S
1.32300 ppm
m/z=476
[M+H]+
Br
200
273
248
121
S
O
OH
HN
N
(E)
+O
The MS/MS fragment of M3-3

(**c**)

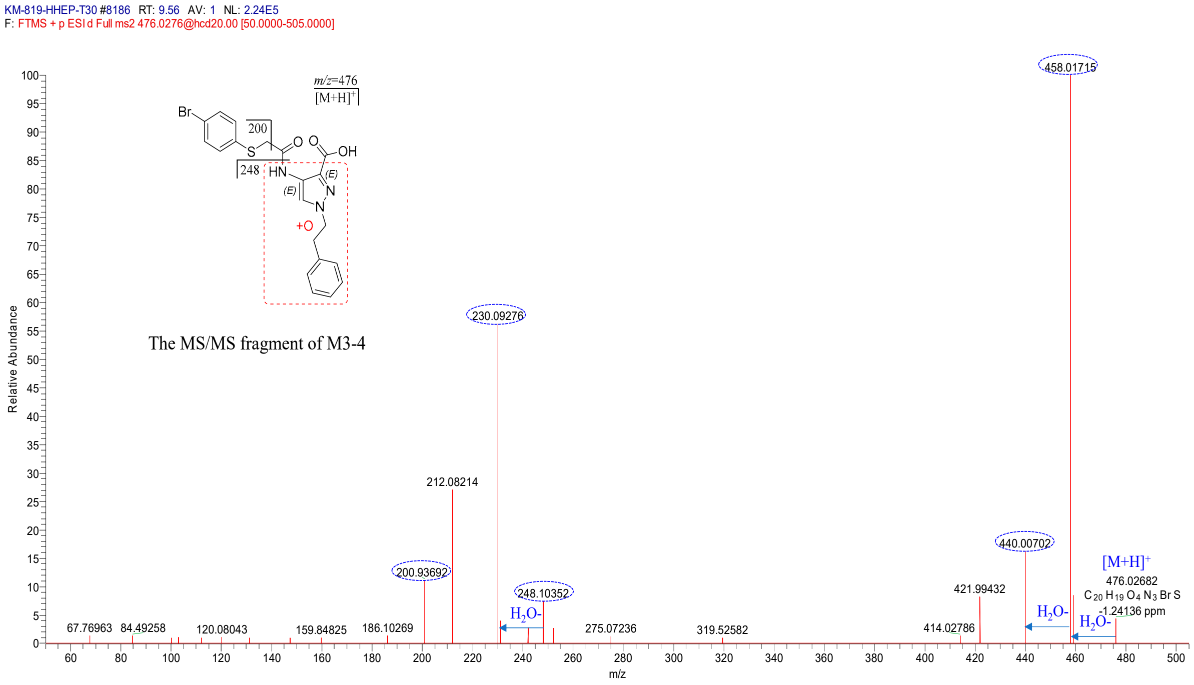

KM-819-HHEP-T30 #8186 RT: 9.56 AV: 1 NL: 2.24E5
F: FTMS + p ESI d Full ms2 476.0276@hcd20.00 [50.0000-505.0000]
Relative Abundance
m/z
458.01715
230.09276
212.08214
200.93692
248.10352
440.00702
421.99432
414.02786
67.76963
84.49258
120.08043
159.84825
186.10269
275.07236
319.52582
H2O-
[M+H]+
476.02682
C20 H19 O4 N3 Br S
-1.24136 ppm
m/z=476
[M+H]+
Br
200
248
S
O
OH
HN
N
(E)
+O
The MS/MS fragment of M3-4

(**d**)

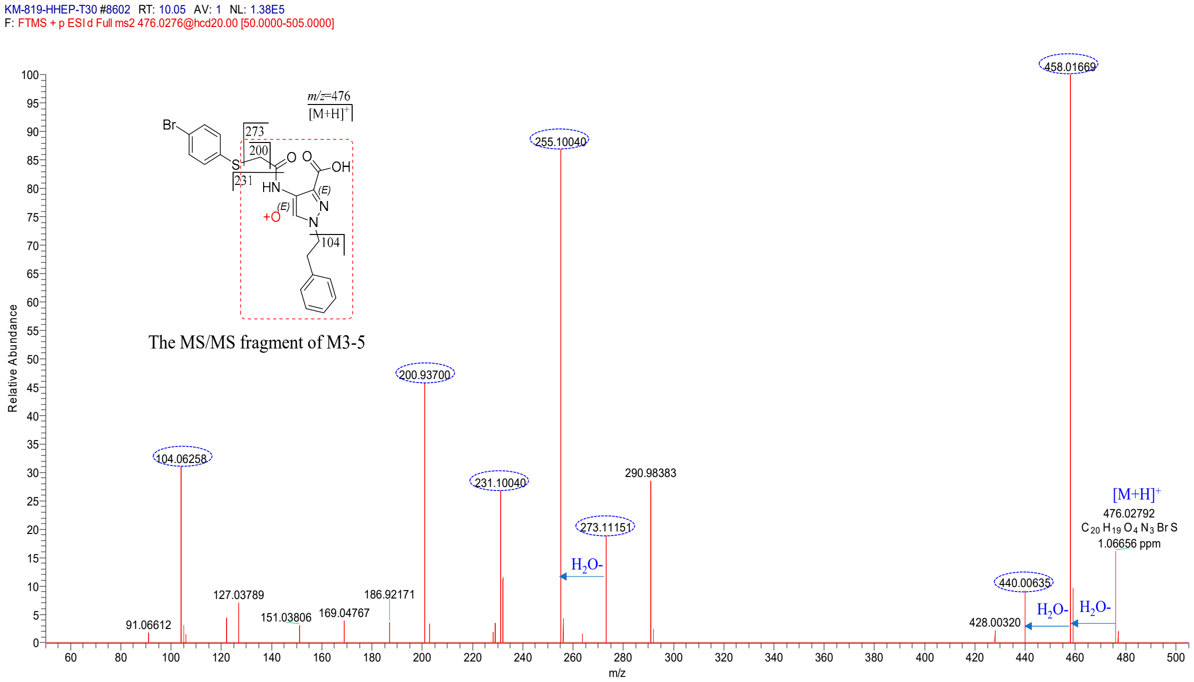

(**e**)

Supplementary Figure S5. MS/MS spectra of (a) M4-1, (b) M4-2, the glucuronidation metabolite of KM-819, and structure elucidation

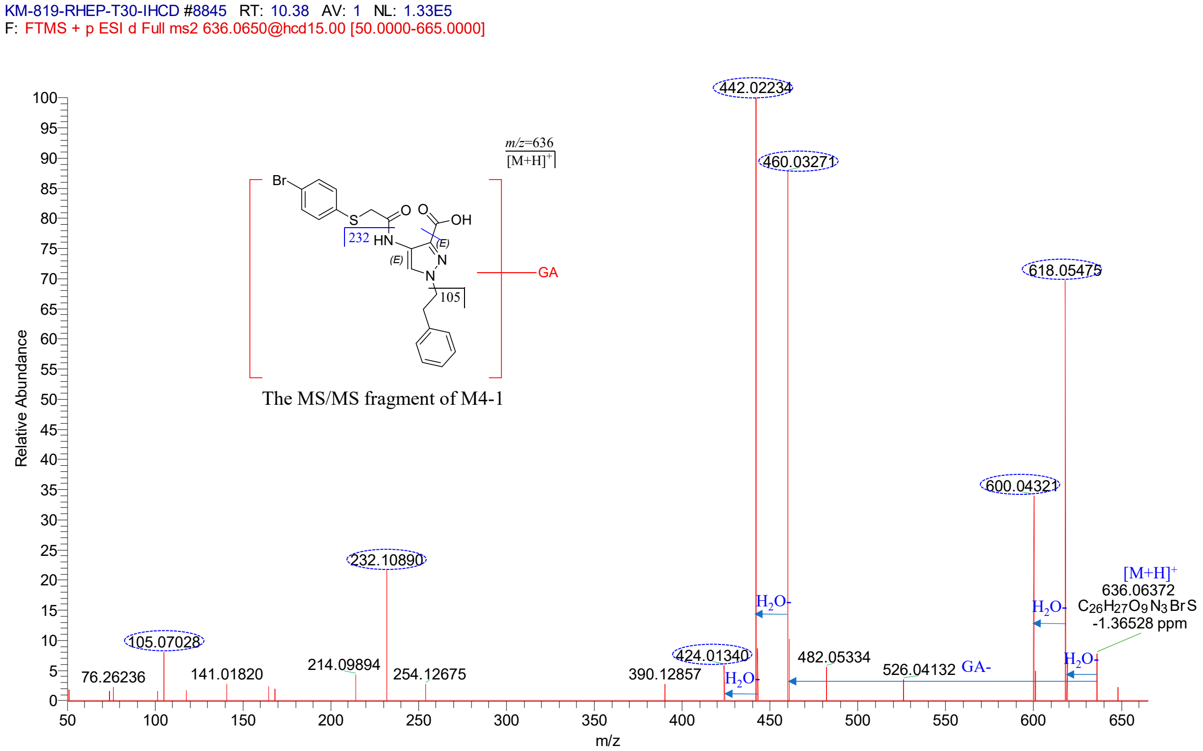

(**a**)

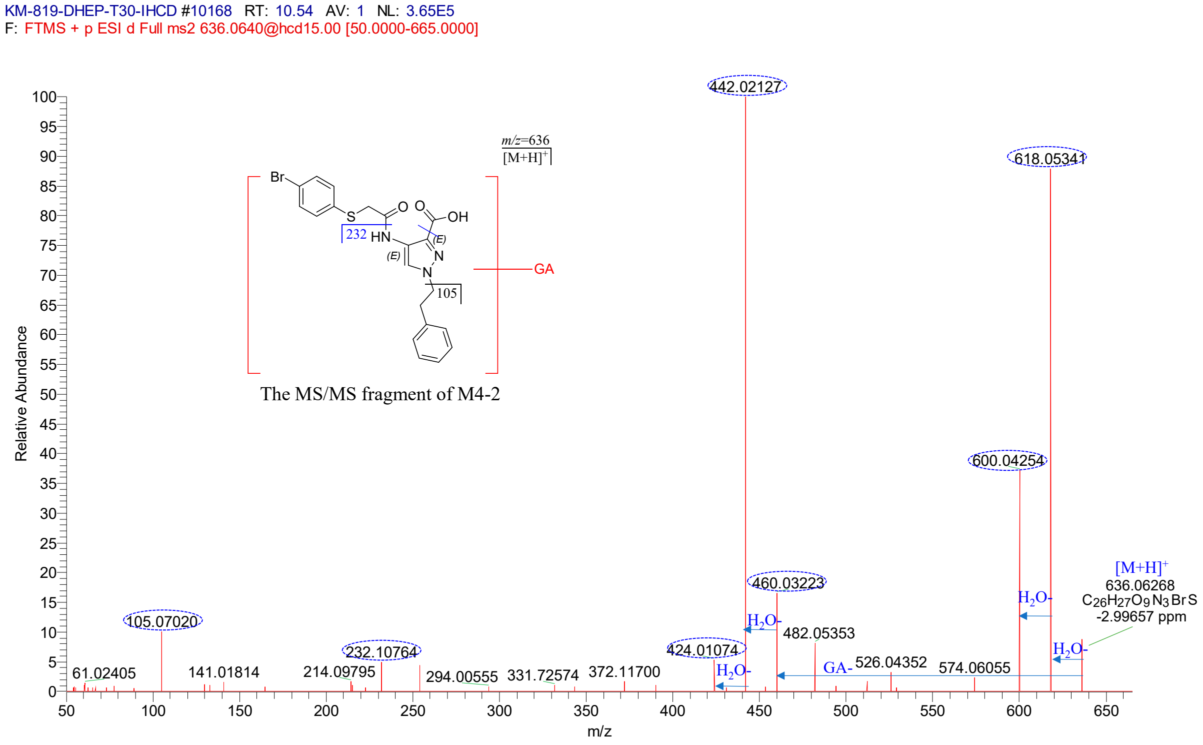

(**b**)

Supplementary Figure S6. MS/MS spectra of M5, the di-oxidation metabolite of KM-819, and structure elucidation

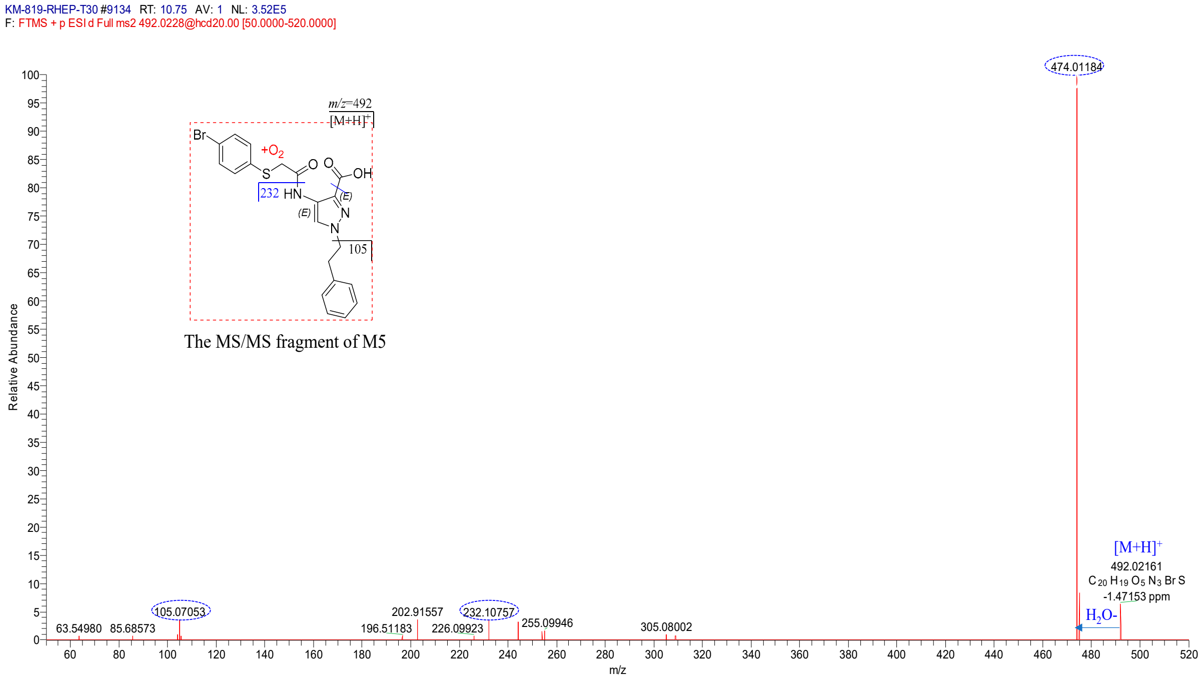

Supplement: Supplementary file 1 [file molecules-29-01004-s001.zip › molecules-2819986-supplementary.pdf]
